# Mitral Valve Replacement via Minithoracotomy Versus Conventional Median Sternotomy in Rheumatic Mitral Valve Disease: A Multicenter Retrospective Study

**DOI:** 10.7759/cureus.86482

**Published:** 2025-06-21

**Authors:** Hicham Kbiri, Rachid Seddiki, Abdellatif Chlouchi, Najib Bouhabba, Amine Meskine, Mourad Ababou, Youssef Qamouss, S Khallkane

**Affiliations:** 1 Cardiothoracic Anesthesiology, Intensive Care Unit, and Emergency, Avicenna Military Hospital, Marrakech, MAR; 2 Anesthesia and Critical Care, Avicenna Training Military Hospital, Marrakesh, MAR; 3 Anesthesiology and Perioperative Medicine, Hopital Cardiologique Louis Pradel, Lyon, FRA; 4 Anesthesiology and Perioperative Medicine, Military Hospital Oued Eddahab, Agadir, MAR; 5 Cardiothoracic Anesthesiology and Reanimation, Mohamed V Training Military Hospital, Rabat, MAR; 6 Cardiothoracic Intensive Care Unit, Marie-Lannelongue Hospital, Paris, FRA; 7 Cardiovascular and Thoracic Anesthesia Department, Hopital Militaire d'Instruction Mohamed V, Rabat, MAR

**Keywords:** conventional median sternotomy, event-free survival, mechanical valve replacement, minimally invasive mitral valve surgery, postoperative complications, retrospective study, rheumatic mitral insufficiency, surgical outcomes

## Abstract

Background and objectives

This study aimed to compare surgical outcomes, early postoperative complications, and midterm recovery in patients with severe rheumatic mitral insufficiency undergoing either minimally invasive cardiac surgery (MICS) or mitral valve replacement via conventional median sternotomy (CMS). While CMS remains the standard approach, MICS has emerged as a less invasive option with potential benefits. However, comparative data in resource-limited settings remain scarce.

Methods

This multicenter retrospective study included 55 adults with severe rheumatic mitral Insufficiency (RMI) who underwent elective mechanical mitral valve replacement between 2020 and 2024 in Morocco. Patients were divided into two groups: 27 received minimally invasive surgery (MICS) via minithoracotomy, and 28 underwent conventional sternotomy (CMS). The primary endpoint was 30-day all-cause mortality. Secondary outcomes included operative times, postoperative complications, intensive care unit (ICU)/hospital stay duration, 12-month functional recovery, valve performance, and event-free survival based on Kaplan-Meier analysis.

Results

Fifty-five patients underwent mechanical mitral valve replacement: 27 via minimally invasive cardiac surgery (MICS) and 28 via conventional median sternotomy (CMS). The 30-day mortality was similar between groups (3.7% vs 3.6%; p = .99). Compared with CMS, MICS was associated with significantly shorter cardiopulmonary bypass (68.3 vs 87.5 minutes; p < .001) and aortic cross-clamp times (54.7 vs 77.1 minutes; p < .001), reduced postoperative pneumonia (0% vs 10.7%; p = .03), and fewer arrhythmias (7.4% vs 39.3%; p = .04). Hospital stay was shorter in the MICS group (6.2 vs 7.3 days; p = .04), with similar ICU duration. At 12 months, both groups showed preserved left ventricular ejection fraction (60.1% vs 58.2%; p = .22) and comparable event-free survival (>90%), without significant differences in valve-related complications.

Conclusions

In this multicenter retrospective study, minimally invasive cardiac surgery (MICS) for severe rheumatic mitral insufficiency was associated with fewer early complications, shorter operative and recovery times, and equivalent 12-month outcomes compared with conventional median sternotomy. These findings support MICS as a safe and effective alternative in appropriately selected patients when performed in experienced surgical centers.

## Introduction

Rheumatic heart disease (RHD), a chronic sequela of group A β-hemolytic streptococcal infections, remains a major cause of cardiovascular morbidity and mortality in low- and middle-income countries (LMICs), in stark contrast to its near-elimination in high-income nations [1]. According to the Global Burden of Disease Study, over 39 million individuals are affected by RHD worldwide, with the vast majority residing in sub-Saharan Africa, South Asia, and parts of the Middle East [2]. Limited access to timely antibiotic prophylaxis, inadequate healthcare infrastructure, and delayed diagnosis contribute to high rates of advanced valvular disease in these regions [3,4]. Among its valvular complications, rheumatic mitral insufficiency (RMI) is a predominant and severe manifestation, frequently presenting at advanced stages with signs of heart failure, pulmonary hypertension, and atrial fibrillation [5]. This study aimed to compare surgical outcomes, early postoperative complications, and midterm recovery in patients with severe rheumatic mitral insufficiency undergoing either minimally invasive cardiac surgery (MICS, n = 27) or mitral valve replacement via conventional median sternotomy (CMS, n = 28).

In contrast to degenerative mitral disease observed in aging populations of the West, RMI in endemic regions often affects younger patients and involves complex anatomical pathology, including leaflet thickening, commissural fusion, and subvalvular fibrosis [6]. These structural complexities increase surgical challenges and impact long-term outcomes. Traditionally, mitral valve surgery has been performed via conventional median sternotomy (CMS), which offers excellent exposure but is associated with increased surgical trauma, longer recovery, and aesthetic concerns [7]. The evolution of minimally invasive cardiac surgery (MICS), particularly through right mini-thoracotomy, offers a less invasive alternative with the potential for reduced perioperative morbidity and accelerated recovery [8].

While MICS has gained traction in mitral valve procedures, its adoption for rheumatic pathology remains debated due to technical difficulties posed by calcified or fibrotic valves and frequent multivalvular involvement [9]. Moreover, evidence comparing MICS and CMS in patients with RMI is limited, especially in real-world LMIC contexts where surgical infrastructure may vary. This multicenter retrospective study aims to compare surgical outcomes, early postoperative complications, and midterm recovery in patients with severe RMI undergoing either MICS or CMS. By leveraging institutional data from high-volume cardiac centers in Morocco, we sought to evaluate the feasibility, safety, and effectiveness of MICS in a resource-limited setting. We hypothesize that, in experienced centers, MICS offers comparable safety with fewer perioperative complications and faster recovery than CMS [10].

## Materials and methods

Study design and setting

This retrospective, multicenter, observational cohort study aimed to compare clinical outcomes between MICS and CMS in patients with severe rheumatic mitral insufficiency (RMI). The investigation was conducted across three high-volume tertiary cardiovascular surgery centers in Morocco, each with established proficiency in both surgical techniques. To ensure transparency, reproducibility, and compliance with journal publication standards, all tables and figures in the manuscript were constructed according to rigorous statistical criteria. All statistical comparisons explicitly report significance thresholds, defined as p < 0.05 for significance and p < 0.001 for high significance. For each analytical comparison, the relevant test statistic (t-value, χ², or exact test result) is reported in the penultimate column of each table to support interpretation of the associated p-value. Finally, each table legend includes the statistical method used, whether an independent t-test, Chi-square, or Fisher’s exact test, ensuring complete methodological transparency.

Study period and data source

Patient data were extracted from institutional surgical databases covering the period from January 1, 2020, to December 1, 2024. Data collection was standardized using harmonized case report forms. Surgical notes, ICU charts, and echocardiographic reports were thoroughly reviewed. Follow-up data were obtained at discharge and at three, six, and 12 months postoperatively. Data formats (e.g., N, %, mean ± SD) were consistently applied and are specified in the table and figure legends.

Preoperative assessment and surgical techniques

All patients underwent a comprehensive preoperative evaluation to assess surgical suitability and plan the operative strategy. This included transthoracic echocardiography (TTE) for characterization of mitral valve pathology, assessment of left ventricular ejection fraction (LVEF), and estimation of pulmonary artery pressures. When available, contrast-enhanced multidetector computed tomography was performed to evaluate thoracic anatomy and peripheral vascular access, particularly to determine the feasibility of a minimally invasive approach.

All patients underwent elective mechanical mitral valve replacement using standardized techniques tailored to the assigned surgical approach: In the MICS group, procedures were performed via a right anterolateral minithoracotomy (4th or 5th intercostal space) with femoral arterial and venous cannulation for cardiopulmonary bypass (CPB). Thoracoscopic assistance was employed to optimize exposure, and myocardial protection was achieved with cold antegrade cardioplegia. In the CMS group, surgery was conducted through a full median sternotomy with central aortic and bicaval venous cannulation.

Both techniques utilized mechanical bileaflet prostheses for valve replacement. Intraoperative transesophageal echocardiography (TEE) was systematically used in all patients to guide prosthesis positioning and to confirm optimal valve function. All procedures were performed by experienced cardiac surgical teams proficient in both approaches. Notably, no conversions from MICS to CMS were required in any patient.

Study outcomes

Primary Outcome

Thirty-day mortality, defined as all-cause mortality occurring within 30 days postoperatively, was reported as a number and percentage (N, %). Comparisons between groups were performed using Fisher’s exact test or the Chi-square test, with the corresponding test statistic and p-value provided. Statistical significance was defined as p < 0.05.

Secondary Outcomes

(a) Cardiopulmonary bypass (CPB) time and aortic cross-clamp time were recorded as mean ± standard deviation (SD) and analyzed using independent t-tests. Relevant t-values and p-values were reported for interpretation. (b) Postoperative complications, including pneumonia, atrial fibrillation, and other arrhythmias, were defined as clinically documented events occurring during hospitalization. These were presented as N (%) and compared between groups using Chi-square or Fisher’s exact test, with corresponding test statistics. (c) ICU and hospital length of stay were measured in days and expressed as mean ± SD. Independent t-tests were used for comparison; for non-normally distributed variables, the Mann-Whitney U test was applied, and U statistics were reported. (d) Left ventricular ejection fraction (LVEF) at 12 months was assessed via transthoracic echocardiography and expressed as mean ± SD. Group differences were analyzed using unpaired t-tests with associated t-values and p-values. (e) Functional recovery was evaluated using the New York Heart Association (NYHA) classification at 12-month follow-up, with improvement or deterioration assessed relative to baseline functional status. (f) Prosthesis-related adverse events-including reoperation, thromboembolic events, structural valve deterioration, recurrent moderate or greater mitral regurgitation, and readmission for heart failure-were summarized as N (%) and verified through follow-up imaging and clinical documentation. Comparisons were conducted using appropriate statistical tests (Chi-square or Fisher’s exact), with test statistics and significance thresholds clearly reported. (g) Event-free survival was defined as survival without any of the following during the 12-month follow-up: all-cause mortality, mitral valve reoperation, recurrent moderate or greater mitral regurgitation, thromboembolic complications, or ≥1 NYHA class deterioration.

Study population and group stratification

A total of 73 adult patients diagnosed with severe rheumatic mitral insufficiency (RMI) were initially identified through surgical databases at three high-volume tertiary cardiovascular centers in Morocco between January 1, 2020, and December 1, 2024. After applying predefined inclusion and exclusion criteria, 55 patients met eligibility requirements and were included in the final analysis.

Inclusion criteria consisted of: (a) age between 18 and 75 years, (b) isolated severe rheumatic mitral regurgitation (regurgitant volume ≥60 mL or effective regurgitant orifice ≥0.4 cm²), (c) preserved left ventricular systolic function (LVEF ≥50%), (d) NYHA functional class III or IV symptoms. Exclusion criteria included: (a) prior cardiac or valvular surgery, (b) LVEF <50% or left ventricular end-systolic diameter >45 mm, (c) multivalvular disease or other structural cardiac abnormalities, (d) active endocarditis, (e) history of stroke or seizure disorders.

**Figure 1:**
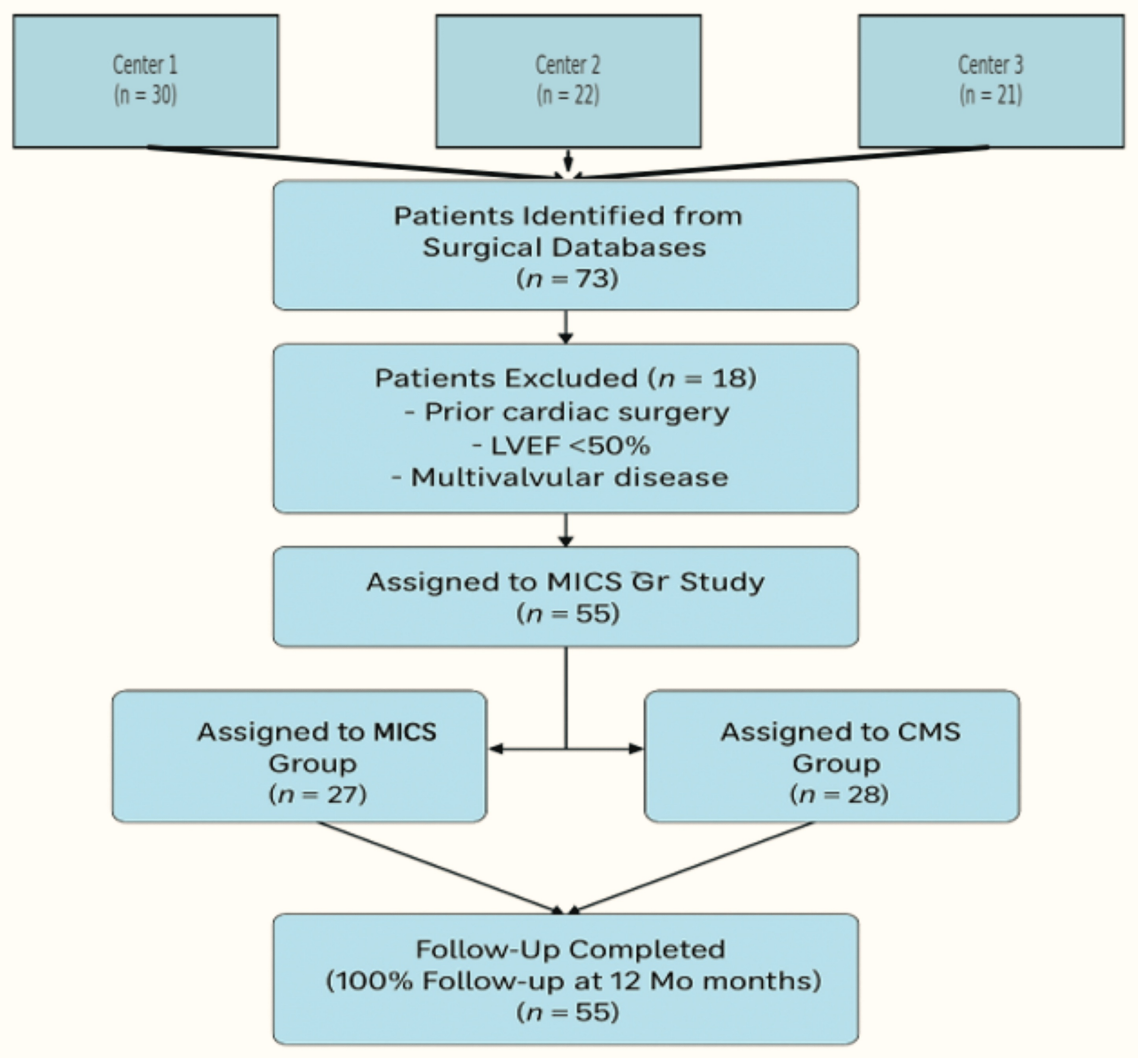
STROBE-adapted patient flow diagram showing screening, exclusion, inclusion, and group allocation across three participating centers STROBE: STrengthening the Reporting of OBservational studies in Epidemiology.

Following eligibility screening, patients were retrospectively stratified into two groups according to the surgical approach: (a) MICS group (n = 27), right anterolateral minithoracotomy with peripheral cardiopulmonary bypass cannulation and (b) CMS group (n = 28), conventional full sternotomy with central aortic and bicaval cannulation. Surgical assignment was determined by institutional protocol and clinical judgment at the time of intervention. A detailed patient flow diagram illustrating eligibility, exclusions, and final group allocation is presented in Figure 1, in accordance with STrengthening the Reporting of OBservational studies in Epidemiology (STROBE) guidelines.

Data collection, quality assurance, and study oversight

Data collection was conducted using standardized case report forms across the three participating centers to ensure methodological consistency. Information was systematically extracted from operative records, intensive care unit (ICU) charts, and echocardiography reports. Follow-up data were obtained at hospital discharge and at three, six, and 12 months postoperatively. A central coordinating team was responsible for data harmonization, discrepancy resolution, and ongoing quality control through routine validation procedures. Oversight was provided by a multidisciplinary steering committee composed of senior cardiac surgeons, anesthesiologists, and clinical methodologists, which ensured strict adherence to the study protocol and institutional standards. An independent data monitoring board conducted periodic audits to verify compliance with ethical and methodological requirements, including the principles of the Declaration of Helsinki. All statistical analyses were performed by a blinded biostatistician, and data confidentiality was rigorously protected through secure data handling protocols.

Statistical analysis

All analyses were conducted according to the intention-to-treat principle. Continuous variables were expressed as mean ± standard deviation or median (interquartile range, IQR), and compared using independent t-tests or Mann-Whitney U tests, as appropriate. Categorical variables were compared using the Chi-square (χ²) test or Fisher’s exact test, depending on distribution and sample size. Event-free survival was assessed using Kaplan-Meier survival analysis, with comparisons performed via the log-rank test. Risk estimates for outcomes were calculated using Cox proportional hazards regression, reported as hazard ratios (HRs) with 95% confidence intervals (CIs). A two-sided p-value < 0.05 was considered statistically significant. All statistical analyses were performed using SPSS software, version 27.0 (IBM Corp., Armonk, NY).

Ethical considerations and data governance

This study was approved by the Institutional Review Board of the Avicenna Military Training Hospital (Approval No. M-70-MAR). All procedures were conducted in accordance with the Declaration of Helsinki and adhered to STROBE guidelines for observational research. Given the retrospective design, the requirement for informed consent was waived. Patient confidentiality was strictly maintained through systematic anonymization and secure data storage, with access to identifiable information limited to the principal investigators. Data governance was ensured through regular internal audits and oversight by an independent monitoring board. A priori protocols were developed and approved by all participating institutional review boards. This study was not eligible for PROSPERO registration, as it does not qualify as a systematic review or meta-analysis.

## Results

Baseline characteristics of the study population

A total of 55 patients with severe rheumatic mitral insufficiency were retrospectively identified and stratified into two groups: 27 patients underwent minimally invasive cardiac surgery (MICS), and 28 underwent conventional median sternotomy (CMS). Baseline demographic and clinical characteristics-including age, sex, body mass index (BMI), and left ventricular ejection fraction (LVEF)-were generally comparable between the groups.

The mean age was 36.4 ± 12.4 years in the MICS group and 35.4 ± 10.9 years in the CMS group (t = 0.37, p = 0.71). The proportion of male patients was similar, 14/27 (51.9%) in MICS vs. 15/28 (53.6%) in CMS (χ² = 0.02, p = 0.88). BMI values were also comparable: 25.9 ± 3.9 in MICS vs. 25.3 ± 3.6 kg/m² in CMS (t = 0.47, p = 0.64). LVEF was 56.8 ± 7.8% in MICS and 54.9 ± 9.2% in CMS (t = 0.85, p = 0.40). Notably, the European System for Cardiac Operative Risk Evaluation (EuroSCORE) II was significantly lower in the MICS group (2.2 ± 1.3%) than in CMS (3.1 ± 1.4%) (t = 2.09, p = 0.04*), indicating a modest reduction in preoperative surgical risk.

Most patients in both groups presented with advanced functional symptoms: 23/27 (85.2%) in MICS and 24/28 (85.7%) in CMS (χ² = 0.003, p = 0.96). Pulmonary hypertension was present in 4/27 (14.8%) of MICS cases vs. 5/28 (17.9%) in CMS (Fisher’s exact test, p = 1.00). For subsequent analysis, event-free survival was defined as survival without any of the following: all-cause mortality, mitral valve reoperation, recurrent moderate or greater mitral regurgitation, thromboembolic complications, or a ≥1 NYHA class deterioration during follow-up.

Continuous variables are reported as mean ± standard deviation (SD), and categorical variables as number (percentage). Statistical significance was defined as p < 0.05 and p < 0.001 for highly significant results. Independent t-tests were used for continuous variable comparisons, while Chi-square or Fisher’s exact tests were used for categorical variables, with the corresponding test statistics and p-values reported to support interpretation and ensure methodological transparency (Table 1).

**Table 1:** Baseline characteristics of the study population LVEF: Left Ventricular Systolic Function, EuroSCORE: European System for Cardiac Operative Risk Evaluation, NYHA: New York Heart Association. MICS: Minimally Invasive Cardiac Surgery, CMS: Conventional Median Sternotomy. Significant p-value < 0.05 is marked with an asterisk (*).

Characteristic	MICS (n = 27)	CMS (n = 28)	Test Statistic	p-value	Test Used
Age, years (mean ± SD)	36.4 ± 12.4	35.4 ± 10.9	t = 0.36	0.71	Unpaired t-test
Male sex, n (%)	14 (51.9%)	15 (53.6%)	χ² = 0.01	0.48	Chi-square test
BMI (kg/m²) (mean ± SD)	25.9 ± 3.9	25.3 ± 3.6	t = 0.49	0.62	Unpaired t-test
LVEF, % (mean ± SD)	56.8 ± 7.8	54.9 ± 9.2	t = 0.70	0.48	Unpaired t-test
EuroSCORE II, % (mean ± SD)	2.2 ± 1.3	3.1 ± 1.4	t = 2.11	0.04*	Unpaired t-test
NYHA class (mean ± SD)	3.3 ± 0.7	3.4 ± 0.5	t = 0.66	0.51	Unpaired t-test
Pulmonary hypertension, n (%)	4 (14.8%)	5 (18%)	Fisher’s exact	0.82	Fisher’s exact test

Primary outcome

Kaplan-Meier Survival Analysis

**Figure 2:**
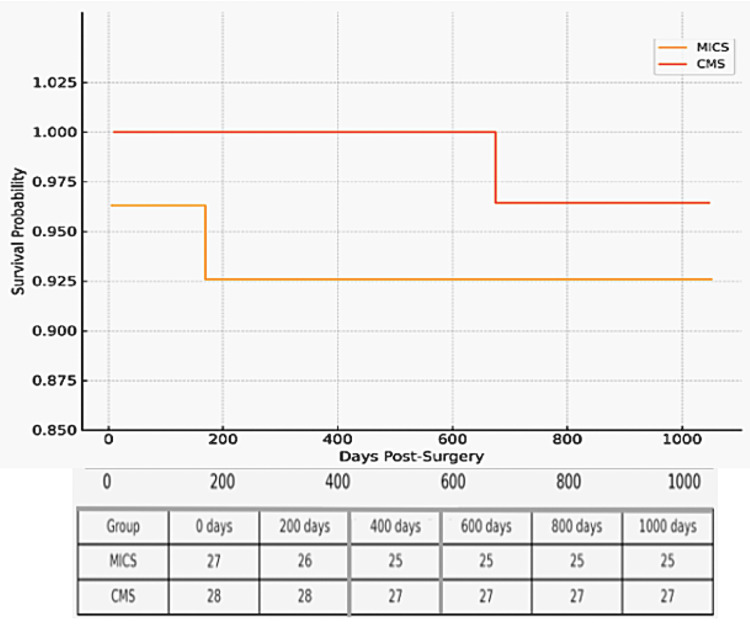
Kaplan–Meier event-free survival curve comparing MICS and CMS groups over 12 months MICS: Minimally Invasive Cardiac Surgery, CMS: Conventional Median Sternotomy. Both groups showed high midterm survival with no statistically significant difference.

A Kaplan-Meier survival analysis was conducted to compare 12-month event-free survival between patients undergoing minimally invasive cardiac surgery (MICS, n = 27) and conventional median sternotomy (CMS, n = 28) for severe rheumatic mitral insufficiency. Event-free survival was defined as the absence of all-cause mortality, mitral valve reoperation, recurrent moderate or greater mitral regurgitation, or a ≥1 NYHA class deterioration. At 12 months, event-free survival was observed in 25/27 patients (92.6%) in the MICS group and 26/28 (92.9%) in the CMS group, with no statistically significant difference between groups (p = 0.94, log-rank test). All values are reported as numbers (percentages). Statistical significance was defined as p < 0.05; p < 0.001 was considered highly significant. Although the MICS group showed a slight numerical advantage, the low rate of adverse events limited the statistical power to detect a meaningful difference. These findings support the non-inferiority of MICS compared to CMS in this population, highlighting its safety and comparable midterm outcomes in experienced centers (Figure 2).

Secondary outcomes

All 55 patients underwent successful mechanical mitral valve replacement without intraoperative conversion from minimally invasive cardiac surgery (MICS) to conventional median sternotomy (CMS). The 30-day mortality rate was identical between groups (1/27, 3.7%) in MICS vs. 3.6% (1/28 in CMS; p = 0.99), and mitral reoperation occurred in one patient in each group (3.7% vs. 3.6%; p = 0.99). Postoperative complications were more favorable in the MICS group: stroke occurred in one CMS patient (3.6%) and none in MICS, while pneumonia was significantly more common in CMS (3/28, 10.7%), compared to none in MICS (p = 0.03*). Arrhythmias were also notably higher in CMS (11/28, 39.3%) versus MICS (2/27, 7.4%; p = 0.04*) (Table 2).

**Table 2:** Postoperative outcomes in MICS vs. CMS groups MICS: Minimally Invasive Cardiac Surgery, CMS: Conventional Median Sternotomy. A p-value < 0.05 was considered statistically significant. Significant values are denoted with an asterisk (*), with highly significant values defined as p < 0.001**.

Outcome	CMS (N=28)	MICS (N=27)	Test Statistic	p-value	Statistical Test Used
Pneumonia	3 (10.7%)	0 (0%)	Fisher	0.03*	Fisher’s exact
Arrhythmia	11 (39.3%)	2 (7.4%)	χ² = 6.45	0.04*	Chi-square
ICU Stay (days)	2.6 ± 1.0	2.1 ± 0.9	t = 1.79	0.07	t-test
Hospital Stay (days)	7.3 ± 2.0	6.2 ± 1.5	t = 2.45	0.04*	t-test

Recovery parameters favored MICS, with a trend toward shorter ICU stay (2.1 ± 0.9 vs. 2.6 ± 1.0 days; p = 0.07) and a significantly reduced hospital stay (6.2 ± 1.5 vs. 7.3 ± 2.0 days; p = 0.04*) (Table 3). At 12-month follow-up, left ventricular ejection fraction (LVEF) remained preserved in both groups without significant difference (60.1 ± 5.9% in MICS vs. 58.2 ± 6.3% in CMS; p = 0.22), and all prosthetic valves maintained satisfactory function. Operative efficiency was superior in MICS, with significantly shorter cardiopulmonary bypass (CPB) time (68.3 ± 8.1 min vs. 87.5 ± 9.6 min; p < 0.001**) and aortic cross-clamp duration (54.7 ± 7.6 min vs. 77.1 ± 8.3 min; p < 0.001**) (Table 3).

**Table 3:** Comparison of CPB and aortic cross-clamp durations between MICS and CMS groups CPB: Cardiopulmonary Bypass, MICS: Minimally Invasive Cardiac Surgery, CMS: Conventional Median Sternotomy. Data are expressed as mean ± standard deviation (SD). Statistical significance was defined as *p < 0.05; **p < 0.001.

Variable	MICS (Mean ± SD)	CMS (Mean ± SD)	Test Used	t-value	p-value
Cardiopulmonary Bypass Duration (min)	68.3 ± 8.2	87.5 ± 9.1	Unpaired t-test	8.89	< 0.001*
Aortic Cross-Clamp Duration (min)	54.7 ± 7.6	77.1 ± 8.4	Unpaired t-test	10.13	< 0.001*

A comprehensive summary of all outcomes, including continuous variables (mean ± standard deviation) and categorical outcomes (n, %), is provided in Table 4. Statistical comparisons were conducted using appropriate tests (t-test, Chi-square, or Fisher’s exact), with significance defined as *p < 0.05 and **p < 0.001.

**Table 4:** Comparative perioperative and follow-up metrics (MICS vs CMS groups) LVEF: left ventricular ejection fraction, MICS: Minimally Invasive Cardiac Surgery, CMS: Conventional Median Sternotomy. Statistical significance was defined as p < 0.05*, with highly significant values defined as p < 0.001**. Appropriate statistical tests (Chi-square, Fisher’s exact, or t-test).

Metric	MICS	CMS	Test Statistic	p-value	Test Used
Cardiopulmonary Bypass Time (min)	68.3 ± 8.2	87.5 ± 9.1	t = 8.23	< 0.001**	Unpaired t-test
Aortic Cross-Clamp Time (min)	54.7 ± 7.6	77.1 ± 8.4	t = 11.4	< 0.001**	Unpaired t-test
Postoperative Pneumonia	0 (0.0%)	3 (10.7%)	Fisher	0.03*	Fisher’s exact test
Postoperative Arrhythmias	2 (7.4%)	11 (39.3%)	χ² = 6.45	0.04*	Chi-square test
ICU Stay (days)	2.1 ± 0.9	2.6 ± 1.0	t = 1.79	0.07	Unpaired t-test
Hospital Stay (days)	6.2 ± 1.5	7.3 ± 2.0	t = 2.45	0.04*	Unpaired t-test
LVEF at 12 Months (%)	60.1 ± 5.9	58.2 ± 6.3	t = 1.25	0.22	Unpaired t-test
30-Day Mortality	1 (3.7%)	1 (3.6%)	Fisher	0.99	Fisher’s exact test
Reoperation Rate	1 (3.7%)	1 (3.6%)	Fisher	0.99	Fisher’s exact test
Stroke	0 (0.0%)	1 (3.6%)	Fisher	0.31	Fisher’s exact test

Functional recovery and echocardiographic follow-up

At 12-month follow-up, transthoracic echocardiography confirmed preserved left ventricular systolic function in both groups. The mean LVEF was 60.1 ± 5.9% in the MICS group and 58.2 ± 6.3% in the CMS group, with no statistically significant difference (t = 1.23, p = 0.22; unpaired t-test). Mechanical prosthetic valve function remained satisfactory in all patients, with only mild residual mitral regurgitation observed in a limited number of cases (MICS: 2/27, 7.4% vs. CMS: 3/28, 10.7%, χ² = 0.16, p = 0.69). Importantly, no episodes of structural valve deterioration, prosthetic dysfunction, or thromboembolic complications were reported in either group during the follow-up period (0/55; 0%).

**Figure 3:**
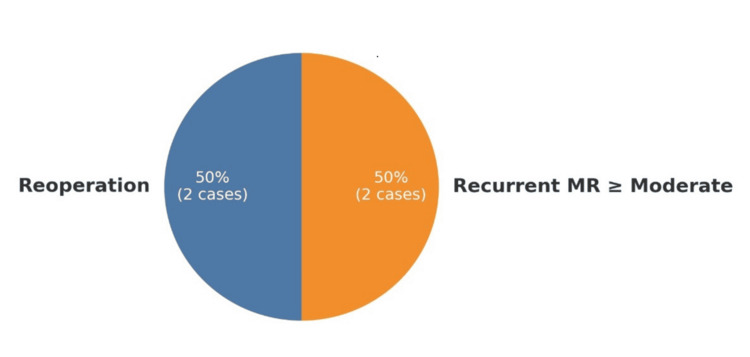
Valve-related adverse events at 12 months follow-up

**Figure 4:**
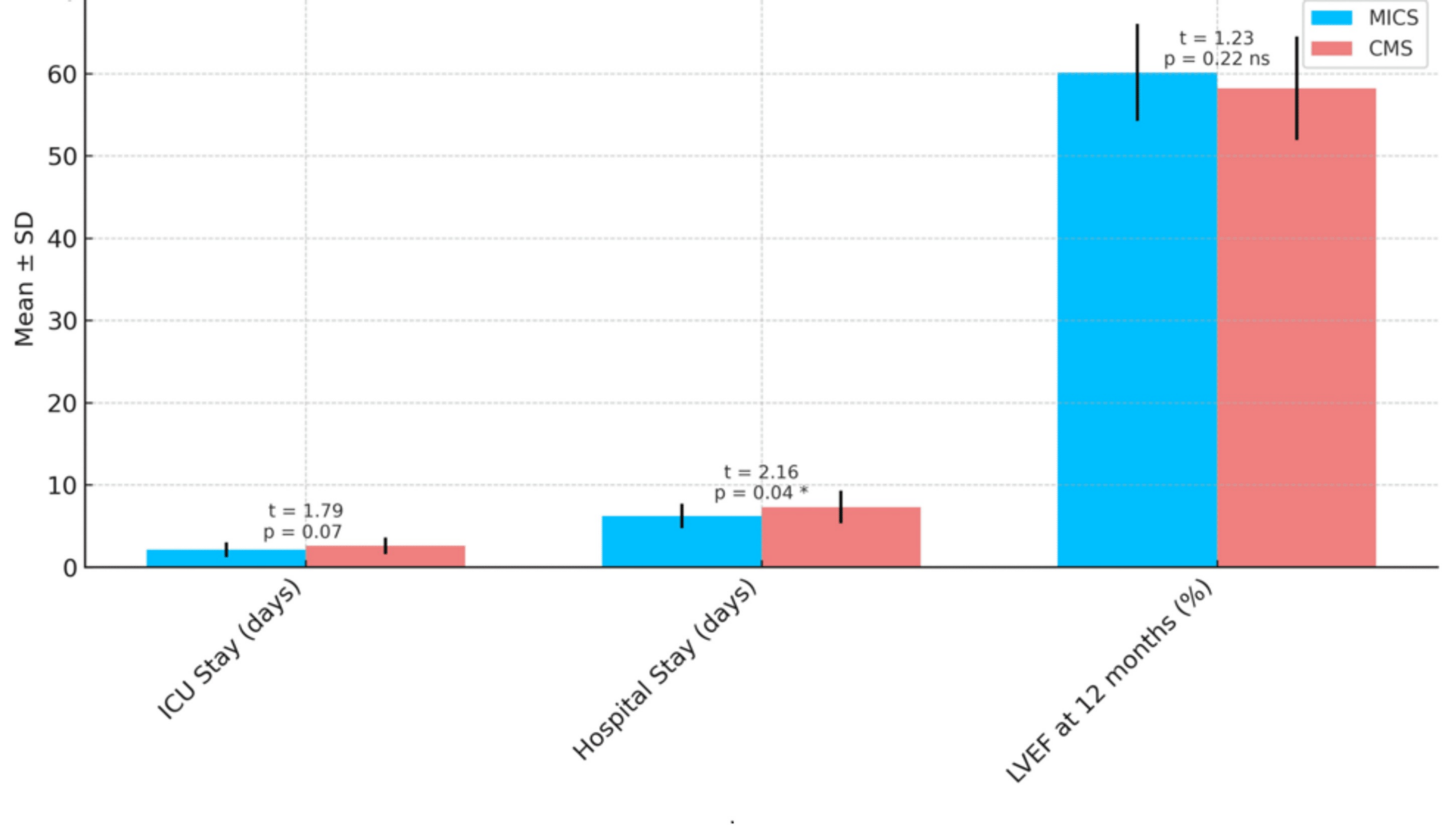
Comparison of ICU stay, hospital stay, and LVEF at 12 months between MICS and CMS groups LVEF: Left Ventricular Ejection Fraction, MICS: Minimally Invasive Cardiac Surgery, CMS: Conventional Median Sternotomy. MICS was associated with shorter recovery times and comparable cardiac function. Data are presented as mean ± standard deviation (SD). Comparisons were performed using unpaired t-tests. Statistical significance was defined as *p < 0.05; **p < 0.001. ns=non-signifigant.

These findings underscore the favorable durability of mechanical prostheses and the equivalent long-term cardiac recovery achieved with both surgical strategies. In addition, perioperative recovery trends continued to favor the minimally invasive approach. The mean ICU stay was 2.1 ± 0.9 days in the MICS group compared to 2.6 ± 1.0 days in the CMS group (t = 1.79, p = 0.07), while the mean hospital stay was significantly shorter in the MICS group (6.2 ± 1.5 vs. 7.3 ± 2.0 days; t = 2.16, p = 0.04), supporting the potential of MICS to optimize postoperative recovery (see Figure 3 for LVEF outcomes and Figure 4 for ICU, hospital stay and LVEF at 12 months comparisons).

## Discussion

This multicenter retrospective study provides evidence that MICS is a safe and effective alternative to conventional median sternotomy CMS for mitral valve replacement in patients with severe rheumatic mitral insufficiency. Although both approaches demonstrated similar 30-day mortality and mitral valve reoperation rates, MICS was associated with significantly fewer postoperative complications, most notably a lower incidence of pneumonia and arrhythmias, as well as shorter hospital stays. These findings align with previously published observational studies and meta-analyses that emphasize the clinical benefits of MICS in reducing perioperative morbidity and expediting recovery in appropriately selected patients [11-13].

In our cohort, no conversions from MICS to CMS occurred, supporting the technical feasibility of MICS even in patients with rheumatic pathology, which is often characterized by complex features such as leaflet calcification, annular remodeling, and subvalvular fibrosis. Our outcomes are consistent with those of Miceli et al. [11] and Borger et al. [12], who demonstrated successful application of MICS in similarly challenging anatomical contexts. Furthermore, Santana et al. [13] reported that MICS reduces respiratory complications, atrial fibrillation, and ICU stay, which aligns with our own findings of a 0% pneumonia rate and significantly fewer arrhythmias in the MICS group (7.4% vs. 39.3%; p = 0.04). [14,15] These complications are not only clinically impactful but also strongly associated with prolonged hospitalization, increased costs, and long-term morbidity [16,17].

Operative efficiency metrics were also favorable for MICS. Cardiopulmonary bypass and aortic cross-clamp times were significantly shorter compared to CMS (both p < 0.001), consistent with literature suggesting that minimally invasive techniques can reduce surgical trauma and hasten postoperative recovery [18-20]. Functional outcomes at 12 months, including preserved left ventricular ejection fraction (LVEF) and >90% event-free survival, confirmed the midterm safety of both approaches [21,22]. The absence of structural valve deterioration or prosthetic dysfunction in either group further supports the procedural durability [23,24].

Postoperative recovery was also enhanced with MICS, as evidenced by shorter ICU and hospital stays and improved NYHA class. These benefits are particularly relevant in low- and middle-income countries (LMICs), where reducing length of stay can ease the burden on limited healthcare infrastructure. Although historically CMS was preferred in RHD due to anatomical complexity [25-27], growing evidence, including our study, demonstrates that with proper patient selection and institutional expertise, MICS is feasible and advantageous, even in this setting [12,28,29].

Multiple meta-analyses have reinforced the benefits of MICS, including decreased transfusion needs, fewer infections, improved cosmesis, and faster return to baseline function, without compromising outcomes [13,20,30]. These advantages are particularly important for younger and female patients, for whom cosmetic results and early return to daily activity are priorities [22]. At one year, both groups demonstrated excellent functional recovery and stability of valve performance, consistent with long-term registry data [31]. By focusing on a younger, rheumatic population often underrepresented in Western trials, this study contributes valuable multicenter data from a real-world LMIC context [32,33]. Kaplan-Meier analysis revealed high event-free survival in both groups, with a slight, non-significant trend favoring MICS. In addition to midterm efficacy, the reduced pericardial adhesions and chest wall trauma associated with MICS may provide advantages in patients who may require future reoperations [25,34-36].

This study has several strengths. It included patients from multiple high-volume cardiac centers with expertise in both techniques, standardized surgical protocols, and consistent data collection. The findings are particularly relevant for LMICs, where rheumatic heart disease is endemic, and optimizing surgical outcomes is of high public health importance. The reproducibility of MICS across all participating centers reinforces its feasibility in rheumatic populations when supported by appropriate infrastructure, imaging, and perfusion techniques.

Nevertheless, important limitations must be acknowledged. As a retrospective observational study conducted in tertiary referral centers, the lack of randomization or propensity score matching limits causal inference and increases the risk of selection bias. Although all procedures were performed in high-volume institutions with harmonized protocols, potential heterogeneity in surgical expertise, perioperative care pathways, and institutional resources may have influenced clinical outcomes. This variability could impact the reproducibility of results in smaller or less experienced settings.

Additionally, the relatively small sample size reduces statistical power, particularly for detecting infrequent adverse events such as thromboembolism or valve-related complications, and limits the generalizability of findings. The exclusion of patients with prior cardiac operations, multivalvular disease, reduced LVEF, or higher operative risk further narrows the applicability of our results to lower-risk, elective surgical populations. This constrained scope should be considered when extrapolating these findings to more complex or urgent clinical scenarios [37].

While the 12-month follow-up provides valuable midterm insights, it does not capture long-term prosthetic durability, thromboembolic risk, or structural valve deterioration. Moreover, the study did not include health economic analyses or patient-reported outcome measures, which are important domains where MICS may offer added value. These limitations collectively highlight the need for future prospective, randomized, and adequately powered multicenter studies to evaluate the long-term safety, effectiveness, and cost-efficiency of MICSs in diverse surgical populations [37].

These findings underscore that, in high-volume centers with appropriate surgical expertise, MICS offers a safe and effective alternative to CMS for rheumatic mitral valve replacement. The reduced complication rates and shorter recovery time associated with MICS highlight its potential to optimize surgical outcomes and resource utilization, particularly in low- and middle-income countries where healthcare capacity is often limited. Importantly, the benefits of MICS are most likely to be realized in carefully selected patients, those with favorable anatomical profiles, preserved ventricular function, and access to specialized perioperative care. As such, strategic implementation of MICS programs in LMICs should prioritize surgeon training, infrastructure readiness, and robust patient selection protocols to ensure sustainable, high-quality care.

## Conclusions

This multicenter retrospective study found that minimally invasive cardiac surgery (MICS) offers comparable 30-day mortality to conventional median sternotomy (CMS) for severe rheumatic mitral insufficiency, while providing advantages such as reduced bypass and cross-clamp times, fewer postoperative complications, and shorter hospital stays. At 12 months, both approaches demonstrated high event-free survival and sustained prosthetic valve function. These results support the safety and efficacy of MICS in selected patients, particularly in experienced centers. Further prospective research is warranted to evaluate long-term outcomes and cost-effectiveness in broader clinical settings.
